# What Actually Works for Activity Recognition in Scenarios with Significant Domain Shift: Lessons Learned from the 2019 and 2020 Sussex-Huawei Challenges †

**DOI:** 10.3390/s22103613

**Published:** 2022-05-10

**Authors:** Stefan Kalabakov, Simon Stankoski, Ivana Kiprijanovska, Andrejaana Andova, Nina Reščič, Vito Janko, Martin Gjoreski, Matjaž Gams, Mitja Luštrek

**Affiliations:** 1 Department of Intelligent Systems, Jožef Stefan Institute, 1000 Ljubljana, Slovenia; stefan.kalabakov@ijs.si (S.K.); simon.stankoski@ijs.si (S.S.); ivana.kiprijanovska@ijs.si (I.K.); andrejaana.andova@ijs.si (A.A.); nina.rescic@ijs.si (N.R.); vito.janko@ijs.si (V.J.); matjaz.gams@ijs.si (M.G.); 2 Jožef Stefan International Postgraduate School, 1000 Ljubljana, Slovenia; 3 Faculty of Electrical Engineering and Information Technologies, Ss. Cyril and Methodius University in Skopje, 1000 Skopje, North Macedonia; 4 Faculty of Informatics, Università della Svizzera Italiana (USI), 6900 Lugano, Switzerland; martin.gjoreski@usi.ch

**Keywords:** activity recognition, machine learning, competition, smartphone, semi-supervised learning, Hidden Markov models

## Abstract

From 2018 to 2021, the Sussex-Huawei Locomotion-Transportation Recognition Challenge presented different scenarios in which participants were tasked with recognizing eight different modes of locomotion and transportation using sensor data from smartphones. In 2019, the main challenge was using sensor data from one location to recognize activities with sensors in another location, while in the following year, the main challenge was using the sensor data of one person to recognize the activities of other persons. We use these two challenge scenarios as a framework in which to analyze the effectiveness of different components of a machine-learning pipeline for activity recognition. We show that: (i) selecting an appropriate (location-specific) portion of the available data for training can improve the F1 score by up to 10 percentage points (p. p.) compared to a more naive approach, (ii) separate models for human locomotion and for transportation in vehicles can yield an increase of roughly 1 p. p., (iii) using semi-supervised learning can, again, yield an increase of roughly 1 p. p., and (iv) temporal smoothing of predictions with Hidden Markov models, when applicable, can bring an improvement of almost 10 p. p. Our experiments also indicate that the usefulness of advanced feature selection techniques and clustering to create person-specific models is inconclusive and should be explored separately in each use-case.

## 1. Introduction

The ubiquity of smartphones, smartwatches and other wearables drives the development of the methods employed to analyze the sensor data that are continuously collected by these devices. Inertial sensors are the most common type, and are well-suited to detecting their users’ activities. However, since many public and proprietary activity-recognition datasets exist, comparing different methods is not easy. This is why the University of Sussex and Huawei prepared a large dataset of locomotion and transportation activities [1], and an activity-recognition competition—the Sussex-Huawei Locomotion-Transportation Recognition Challenge (SHL Challenge)—has been organized annually from 2018 to 2021, and may continue in the future. Its objective is to recognize locomotion and transportation activities: walking, running, cycling, and travelling by car, bus, train and the underground.

The 2018 SHL Challenge [2]—the first one—presented a straightforward activity-recognition problem suitable for classical activity-recognition approaches. The competition was linked with the Human Activity Sensing Corpus and Applications (HASCA) workshop at the UbiComp conference, which is one of the larger workshops at the leading conference on ubiquitous computing. This is where the competitors’ approaches were presented and the winners were announced. To keep the SHL Challenge interesting, the 2019 and 2020 editions introduced several additions. In 2019 [3], the training data were obtained from phones placed in the pocket and backpack, while the test data were collected from phones in the hand, and there was only a small validation set that included hand data. In 2020 [4], the training data were obtained from one person, while the test data were collected from two other persons, and the small validation set included these two persons. In addition, the location of the phone in the test data was unknown. This encouraged the exploration of new approaches involving transfer learning and domain adaptation. The 2021 edition moved away from inertial sensors and focused on location and radio data. Our team won the 2018 and 2019 SHL Challenges, and placed third in the 2020 Challenge. The solution for the 2018 competition is described in our previous paper [5], whereas this paper describes our 2019 and 2020 solutions.

This paper first explores the question of how to utilize a large training set that is somewhat different from the test set, and a validation set that is smaller but more similar to the test set. This problem is also known as a domain shift (or distributional shift) [6]. While domain shifts are common in practical applications of machine learning, conventional machine-learning models often fail. The paper also investigates whether all classes should be recognized by the same model, or whether separate models should be used to recognize human locomotion and transportation in vehicles. Furthermore, for the 2020 dataset, the unknown test phone location is recognized before an attempt is made to distinguish the users in the validation and test data. Finally, we employ two methods that are generally useful in activity recognition: smoothing with a Hidden Markov Model (HMM) and semi-supervised learning. The paper shows the extent to which the recognition accuracy benefits from each of these steps for both the 2019 and 2020 datasets.

The rest of the paper is organized as follows. Section 2 briefly presents related work on activity recognition, particularly related to the domain shift. Section 3 describes the 2019 and 2020 SHL Challenge datasets. Section 4 explains the data pre-processing and feature extraction. Section 5 describes the core of the used methods. Section 6 provides the experimental results. Finally, Section 7 concludes the paper with a summary, lessons learned and avenues for further research.

## 2. Related Work

Today’s smartphones are powerful devices equipped with a wide variety of sensors, which allow for researchers and developers to infer much about the user’s context, such as the location or the activity in which they are engaged. The usefulness of such information has made activity recognition with wearable sensors a thriving research area in the past decade. Much of the work regarding activity recognition has focused on healthcare applications, such as care for the elderly [7], and chronic disease management [8], although the user’s activity can be exploited in quite diverse ways (e.g., for music selection [9]). Our paper addresses the methodology in general and does not focus on any particular use-case. The activities that are to be recognized can also be diverse, ranging from broad ones such as walking [10], which is the type addressed in our paper, to more specific ones, such as walking up- and downstairs and on slopes (e.g., [11]).

One area of activity recognition that has not enjoyed significant attention until recent years is the detection of transportation modes [12,13]. However, with the introduction of the SHL Dataset [1] and SHL challenges, many new approaches to locomotion and transportation mode recognition have been proposed. In terms of machine learning algorithms, following the example of earlier work [14,15,16], these approaches have leaned toward the use of deep learning. Some of the best results posted during the challenges were achieved using the MultiResNet architecture [5], IndRNN architecture [17] and the DenseNetX architecture [18]. Nevertheless, these challenges have also shown that, alhough classical machine learning pipelines require domain knowledge for meaningful feature extraction, selection and tuning [19], they are still very competitive and sometimes produce better results than the aforementioned deep learning methods. An additional advantage is that they can be more easily understood and adapted to the specifics of new activity recognition scenarios, which is why they are the chosen approach in this paper.

Regardless of the choice to use deep or classical machine learning, activity recognition pipelines are prone to difficulties when performing on data that slightly differ to those used in the training process [20], and the SHL challenges have tried to focus the research community’s attention on that problem. This is why, based on our work in the aforementioned challenges, in this paper, we explore the effectiveness of different classic machine learning pipeline components in combating the problem of domain shift.

Methods to address the domain shift have existed for a long time [21] and have many applications [22,23,24,25,26,27,28]. The primary objective of these methods is to find ways to apply a model trained in one or more “source domains” to a different (but related) “target domain”. The source and target domains all have the same feature space, but different distributions. For example, data from sensors placed on the chest and the leg differ significantly from each other. Additionally, significant differences can be found in the data of two people (each person has a specific way of walking, running, etc.). In deep learning, a widely used method to address the domain shift is to train a deep neural network on the source dataset, and to fine-tune it on the target dataset [29]. In classical machine learning, addressing the domain shift is less straightforward. This can be carried out by transforming the feature spaces so that the source and target distributions better fit, either by changing the feature representation according to some explicit distribution distance metric [30] or by weighting the features [31]. An alternative approach is to weight the instances according to their relevance to the target domain [32].

As mentioned previously, in the 2019 SHL Challenge, the focus was on the use of data from different sensor locations to build a model for another location. This can be regarded as a domain shift problem, where the source and target domains differ in the sensor location. A simple way of addressing this in the related work was to train a general activity recognition model using sensor data from different body locations [33]. Khan et al. [34] used both the location of the smartphone and the activity category as separate class labels, and thus forced the model to consider the location of the smartphone when classifying the activity. Shi et al. [35] proposed a framework that first extracts location features and detects the smartphone location, and then performs location-specific activity recognition. In our work, we also detected the smartphone location, and we experimented with general and location-specific activity recognition models, and with the selection of transferable features (which is an extreme form of feature-weighting).

The 2020 SHL Challenge presented a domain shift problem where the source domain was one person and the target domain was two other persons. Hachiya et al. [36] proposed a cross-person activity recognition model, where they solved the domain shift problem by weighting the samples from the source dataset. Venkatesan et al. [37] also used a sample weighting approach, and, in addition, proposed a new cost-sensitive boosting algorithm that they used to weight the training samples. In our work, simply combining the source and target data proved to be adequate, but we did experiment with the selection of transferable features.

Another potential solution to both of these domain shift problems is semi-supervised learning [38]. The idea of semi-supervised learning is that unlabelled data from the target domain, which are often plentiful, are used to improve machine-learning models. There are several pieces of related work that highlight the benefits of semi-supervised learning with classic ML algorithms when labeled (target) datasets are small [39,40,41]. Although Guan et al. [39] presented an interesting take on the co-training approach, which circumvents the need for two disjoint feature sets that this approach otherwise requires, in our work we decided to focus on the more fundamental self-training approach. We aimed to reduce the number of models that play a role in the learning process, since semi-supervised learning was another step in an already complex pipeline, and because the performance of co-training and self-training were shown to be similar when using acceleration data [41].

In addition to the domain shift, another difficulty in the 2020 SHL Challenge was that we did not know which sample from the test data belonged to which person. However, from related work [20], we know that activity recognition models usually work better when personalized. Thus, to improve the performance of our pipeline, we clustered the sensor data into two clusters, which were intended to correspond to the two test persons. There are few related works on this topic, and we relied on a similar clustering to that proposed by Vo et al. [42]. A similar approach was proposed by Kose et al. [43], where k-nearest neighbors was used to create smaller training sets for each activity, and classification was performed based on these reduced sets. We came up with a way of selecing the clustering features that were tailored to the 2020 SHL Challenge.

In addition to all of this, we also explored the effectiveness of post-processing the pipeline predictions with Hidden Markov Models. This approach was successfully used in related work (e.g., [44]). However, we could exploit its interaction with semi-supervised learning as having two predictions—regular and smoothed—which provides interesting opportunities to decide what labels to include in which training data. We are not aware of related work addressing this issue.

This paper is based on the workshop submissions that accompanied our SHL Challenge entries in 2019 [45] and 2020 [46]. However, the methodology is significantly refined and harmonized across the datasets of both years to the degree this is feasible, and the results are more extensively analyzed.

## 3. SHL Challenge Datasets

Both the 2019 and 2020 SHL challenges were centered on the SHL Dataset [1,47], one of the largest publicly available datasets for activity recognition. Although there are several popular publicly available datasets for activity recognition, such as MHEALTH [48,49], UCI-HAR [50], OPPORTUNITY [51], PAMAP2 [52] and Skoda [53], very few of them include as rich a combination of sensor modalities, sensor placements and high sampling frequency as the SHL Dataset [1,47]. Most of the aforementioned datasets only provide data that were gathered by Inertial Measurement Units (IMUs), while the subset of the SHL Dataset used in the challenges and this study also provides orientation and air pressure data. Additionally, most of the popular activity recognition datasets fix their IMUs in place, at locations such as the chest, ankle or at various positions on the arms. While these placements are certainly good choices when analyzing the motion of different body parts, the SHL Dataset allows us to analyze data from the locations (hips, torso, bag, and hand) that are arguably the most realistic smartphone placements during day-to-day activities. Furthermore, in the SHL Dataset, the position of the smartphone at each of those locations is not fixed and can assume any orientation, which makes for a more accurate representation of real-life scenarios. Finally, the SHL dataset also allows us to explore an aspect of activity recognition that is not very common, i.e., the recognition of different modes of transportation. As was previously mentioned, the goal of the dataset, as well as the 2019 and the 2020 SHL Challenges, is to allow for the recognition of eight modes of locomotion and transportation (car, bus, train, subway, walking, running, biking, or being stationary).

The SHL dataset was originally recorded using four smartphones worn at different on-body locations (*hand*, *hips*, *torso*, *bag*). The smartphone model that was used in the data collection procedure is the Huawei Mate 9 Android phone. For the actual recording and annotation of data, the authors used their own Android data logging application (Available online: https://github.com/sussexwearlab/DataLogger accessed on 1 May 2022) and an in-house annotation tool [54]. From each smartphone, the authors recorded data from 15 different sensor modalities [47].

In total, three different users participated in the data collection procedure; however, information such as their age and gender was not made public. In terms of how the data collection was performed, the users were encouraged to go about their daily activities as if the data collection process was not happening. However, they were given a weekly goal of how many hours of data they had to collect per activity.

Even though the original dataset contains 2812 h of data, only a subset was released for the purpose of the activity recognition challenges. This subset contained data sampled at a frequency of 100 Hz and, instead of all 15 available sensor modalities, included the following subset of sensors: triaxial acceleration, triaxial gravity, triaxial gyroscope, triaxial linear acceleration, triaxial magnetic field, orientation as quaternions, and air pressure. GPS, WiFi, and other sensor data that could be used to identify the location of the user were omitted. The data were segmented by the organizers using five-second windows, while labels were provided for each sample. The distribution of the activities in the *SHL-Train* and *SHL-Validation* data was quite uniform, except for the running activity, which was understandably under-represented.

In both years, the provided data came in three sets: *train*, *validation*, *test*. We used the *SHL* prefix to distinguish the original datasets from the datasets used in the experiments. The need for such prefixes arose from the fact that the training dataset in an experiment could be, for example, a combination of all instances from the *SHL-Train* and three-quarters of the instances from the *SHL-Validation* set.

Details about how these data were split in the 2019 and 2020 SHL Challenges, with respect to sensor modalities, sensor locations and users, is provided in the following subsections.

### 3.1. 2019 Dataset

The *SHL-Train* set contained the sensor data streams from three phone locations (*bag*, *hips*, and *torso*). This set was the largest and consisted of 59 days of data. In the *SHL-Validation* set, data were provided from all four locations, including the *hand* phone location, for a period of only three days. Last, the *SHL-Test* set contained only the *hand* location and was unlabeled; labeling it correctly was the competition’s goal. This set contained 20 days of data summarized in the upper part of Table 1. All three sets of data, the *SHL-Train*, *SHL-Validation*, and *SHL-Test* set, contained data collected by a single user.

### 3.2. 2020 Dataset

The *SHL-Train* set was the largest and was composed of data from one user (User 1) and all four phone locations. The *SHL-Validation* set was relatively much smaller and contained mixed data from the other two users (User 2 and User 3) for all four locations. Finally, the *SHL-Test* set contained data from User 2 and 3 and one unknown phone location. This set was unlabeled, and correctly labeling it was the competition’s objective. Overall, the challenge data comprised 4 × 59 days of *SHL-Train* data (59 days of data for each of the four locations), 4 × 6 days of *SHL-Validation* data, and 40 days of *SHL-Test* data, as summarized in the lower part of Table 1.

### 3.3. Reconstruction of the Order of the Data

The *SHL-Test* data from 2019, as provided by the organizers, was split into five-second segments, which were then randomly shuffled. To be able to incorporate the temporal information as a post-processing step, however, we needed to provide data in the correct order. Thus, we performed steps to algorithmically re-order the dataset.

The key insight for doing this was that the end of one data segment best matches the beginning of another segment—presumably the one that followed it in the original ordering. This gives rise to the following algorithm: (1) Choose a random segment *x*, and (2) find the five-second segment that has the minimal distance from its first sensor readings to the last sensor readings of *x*. The distance used is the Euclidean distance, with different sensors weighted using empirically determined weights. (3) If the minimal distance is below a threshold value, join the segments and then repeat this process, using the newly created segment instead of the random one. If it is not, choose a new random segment to repeat the process (this would only happen if there are omissions or missing data in the original dataset). This procedure is summarized in Algorithms 1 and 2.

The output of this algorithm consists of long strings of joined five-second segments that are locally ordered, but the ordering of these strings is unknown. This partial and local ordering, however, is sufficient for our purposes.

When trying to apply the same algorithm to the *Test* data from 2020, we obtained strings composed of only one segment. These results implied that the algorithm did not find any segments that sequentially followed each other. Presumably, this is because, in 2020, when providing the data, the organizers excluded some of the data between two sequential window frames.

To re-order the data, we tried to train machine-learning models that show whether two segments are sequential, although there are some missing data between them. Since we did not have any segments that were longer than five seconds from the 2020 challenge, we used the obtained segments from 2019 as training data. For features, we used two sensor readings. If the time between the two sensor readings was less than five seconds, we labelled the sample as sequential; otherwise, we labelled it as non-sequential. For the machine-learning model, we used Random Forest. However, the model showed poor classification accuracy; therefore, we abandoned the idea of re-ordering the data in the 2020 challenge.
**Algorithm 1** REORDER-DATA**Input: **windows, thresholdallOrderedSequences←∅index←0**while **length(windows)≠0**do**    create array orderedSequence    distance←0    current← random(windows)    **while** distance<threshold **do**        orderedSequence←windows[index]        windows←windows\{current}        current,distance← CLOSEST-WINDOW(current,window)    **end while**    insert orderedSequence into allOrderedSequences**end while****Output: allOrderedSequences**

**Algorithm 2** CLOSEST-WINDOW
**Input: **currentWindow, windows


lastRecording←currentWindow[length(currentWindow)]



closestWindow←windows[1]



distance←∞



i←2


**while** i<length(windows) **do**
    **if** EuclideanDistance(lastRecording,windows[i][1])<distance **then**        distance←EuclideanDistance(lastRecording,windows[i][1])        closestWindow←windows[i]    **end if**
**end while**


**Output: closestWindow,distance**



## 4. Pre-Processing and Features

In this paper, we opted to use classical machine learning, as it yielded better results than our internal deep learning experiments. To employ this method, we first needed to pre-process the data and calculate a large body of features which would describe these data.

The pre-processing started by lowering the sampling frequency of the sensors to 50 Hz to lessen the computational load, mainly because our previous work [5] showed that such a decision does not have any negative performance impacts on a classic ML pipeline. We then filtered the data to remove noise and emphasize the parts of the signal in which the activities performed by the user might be most visible. In the next step, virtual sensor streams were created to derive the information that is part of the sensor signals but might not be visible at first glance. Finally, the data were segmented into windows to prepare for feature extraction.

In terms of feature extraction, our approach was to include a large body of features that have proven to be helpful in our past and related work and then let automatic feature selection select only those most suitable for the problem at hand. We chose this approach to not rely on human feature selection, which is often difficult to justify and requires significant domain expertise.

### 4.1. Filtering

Raw sensor data were iltered using low-pass and band-pass filters. The sensor data were filtered using a low pass infinite-impulse-response (IIR) filter and a band-pass filter, which was a combination of high- and low-pass IIR filters. The smoothing factors for IIRs were determined experimentally.

### 4.2. Virtual Data Streams

From these original sensor streams, it is possible to derive additional sensor streams that are useful for activity recognition. All subsequent steps treat these derived sensor streams in the same manner as the original ones.

The first derived sensor stream is the magnitude of the data. This was calculated for all the data from the triaxial sensors (acceleration, linear acceleration, gravity, magnetic field, and angular velocity). The additional derived sensor streams were Euler angles, which were calculated from the quarternion data. Although using quaternions is better for avoiding a “gimbal-lock”, Euler angles are more suitable as features, as each one is informative on its own. In contrast, quaternions need to be analyzed as a set of values.

### 4.3. Windowing

To extract features from sensor data, data streams need to be segmented into windows. Our previous work [5] on the 2018 SHL Challenge found that using longer windows yields better results—presumably due to the infrequent activity transitions. In the 2019 and 2020 challenges, organizers provided data in five-second segments (see Section 3.3), which was already much shorter than the one minute segments used in 2018. Therefore, we used these five-second segments directly as windows for feature extraction.

### 4.4. Features

To use classical machine learning, we calculated the features of each five-second window of data. Labels were determined for each window as the most frequent per-sample label in a given window. The calculated features can be roughly categorized into the frequency domain and the time domain. The following two subsections describe each category. The exact implementation of each feature is available in our open-source feature extraction library [55]. Altogether, 1124 features were calculated.

#### 4.4.1. Frequency Domain Features

These features were calculated using the power spectral density (PSD) of the signal, based on the fast Fourier transform (FFT). PSD characterizes the frequency content of a given signal and can be estimated using several techniques. Two of the most widely used and commonly considered are a simple periodogram, which is obtained by taking the squared-magnitude of the FFT components, and Welch’s method, which is a bit more complex but superior to periodogram.

In our work, we used the Welch’s method to obtain the PSD. We implemented the same frequency-domain features as in the previous competitions [45,56]—the three largest magnitudes of the FFT components, entropy of the the normalized FFT components and their energy.

#### 4.4.2. Time-Domain Features

We used time-domain features that were proven to be successful in [57] and previously won competitions [45,56]. These features were designed for accelerometer data, and most of them were calculated based only on the acceleration (and its derived) data streams. Some of the features were also calculated based on the gyroscope data streams; however, some features, such as *linear velocity*, were left out as they have no semantic interpretation when calculated based on non-acceleration data.

We also extracted a subset of time-domain features from the tsfresh library that were not included in the previous set of expert features. These features were: the signal minimum, maximum, standard deviation, the number of times the signal is above/below its mean, the signal’s mean change/absolute change, and its different autocorrelations (correlations of the signal with a delayed version of itself, for three different delays).

## 5. Methods for Activity Recognition

This section describes the methods that we developed for the 2019 and the 2020 SHL Challenges. The methods used for both these years include vehicle-specific models, feature selection, and semi-supervised learning. We consider these joint components to be more general for activity recognition, as they proved to be useful in both years. In addition to the joint components, the 2019 pipeline also included temporal prediction smoothing using HMMs, while the 2020 pipeline included the recognition of unknown locations and person clustering. The details of each component, and how they were combined in specific machine learning (ML) pipelines in 2019 and 2020, are presented in the following subsections.

### 5.1. Recognition of Unknown Location (2020)

We investigated whether it is possible to build a classification model to detect the phone’s location, which can be used to explore the benefits of location-dependent activity recognition models.

The placement of the sensors on the body has a significant impact on the sensor signals acquired while a person is performing dynamic activities, such as walking and running. This is mostly due to the fact that different body parts have varied degrees of freedom and, consequently, different movement patterns. Further, the signals recorded while a person is using one of the several considered modes of transportation (car, subway, bus, or train) are extremely similar regardless of the placement of the sensors on the body, as the body itself is usually not in motion. With this consideration, we carried out the phone location detection in two steps: (i) detection of dynamic activities, i.e., walking and running; and (ii) detection of phone location based on the identified instances of dynamic activities.

**Figure 1:**
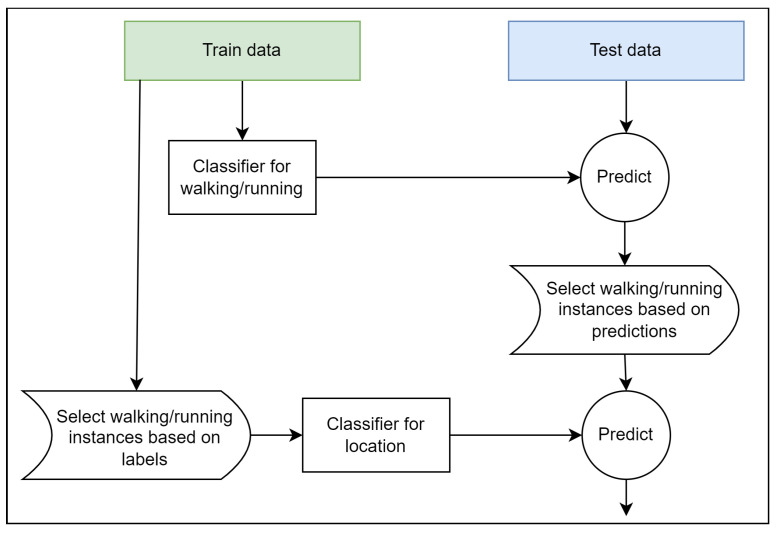
Pipeline for recognizing the unknown phone location of the Test set.

In the first step, sensor data from all eight activities, recorded for all four locations (*torso*, *hips*, *hand*, and *bag*), were used to train a classification model that can identify the instances belonging to dynamic activities, i.e., walking and running. The task was formulated as a binary classification task. In the second step, only instances referring to the walking and running activity were used to train a classification model that can identify where the phone is placed on the body. The full pipeline for recognizing the Test set’s unknown location is illustrated in Figure 1.

The same feature set was utilized to train both classification models (for walking/running detection and phone location detection), and contained all the extracted features (see Section 4.2). Both walking/running detection and phone location detection employed the Random Forest algorithm.

### 5.2. Vehicle-Specific Models (2019 and 2020)

Having separate models for different transportation modes allows for them to be tailored to the specific modes and, thus, perform better. The reasoning for this is based on the level of human activity associated with the specific transportation modes, namely dynamic activities (running, walking, and biking) and static activities (travelling by bus, car, subway, or train or being stationary). Furthermore, having separate models provides the opportunity to obtain different sets of training data for each model, which can be especially useful for vehicle-specific classes.

Motivated by the assumption that different locations (2019) or persons (2020) matter less in vehicles-specific classes, we explored whether combining the data from the *SHL-Train* and *SHL-Validation* sets for training might improve the models. The reasoning behind why different locations or persons might matter less in vehicles is explained for each year separately.

The data collected from devices placed on different body part are similar when a vehicle-specific activity is performed (travelling by car, bus, train, or subway). This is primarily true because, when resting in a vehicle, all the body locations are subject to similar vehicle vibrations regardless of where the phone is placed.

The same is true for different participants. For the vehicle-specific transportation modes, the data for different participants are similar. This can be explained by the fact that, when using these transportation modes, the user is mostly static. Therefore, the main factor that affects the sensors’ signals is the vehicle vibrations, which are not dependent on the specific user.

For the non-vehicle activities, the reasoning is the opposite. This is mainly true because these activities depend on human movement, which can vary greatly between different persons. Additionally, the human movement affects the data from each device location differently. As a result, we explored if using only the *SHL-Validation* set for training, which has same characteristics as the *SHL-Test* set (same device location for 2019 and same participant for 2020), can improve the performance.

### 5.3. Person Clustering (2020)

We explored the possibility of using clustering to divide the *SHL-Validation* and *SHL-Test* sets into two subsets each, such that each subset would contain data from only one user. This would allow for us to build user-specific models and, hopefully, increase the performance of our model.

By using the K-means algorithm, the *SHL-Validation* data were first clustered. We completed this with all the features and with the features that were expected to best distinguish between the users. To select these features, we used models trained to distinguish 25% of the *SHL-Train* set (which included User 1) and the entire *SHL-Validation* (which included User 2 and 3). When training the models, we compared different feature selection algorithms—recursive feature elimination (RFE), the wrapper method, and the first 50 most important features for each location according to Random Forest’s internal feature importance measure.

Next, the clusters were modified so that all the consecutive samples belonged to the same cluster, using the algorithm for reconstructing the order of the data described in Section 3.3. Using the smoothed labels, we built a classification model that could be used on the unlabeled *SHL-Test* set.

To include the person clustering in our pipeline, we attempted to combine this with the vehicle-specific procedure described in Section 6.4. However, this resulted in the further subdivision of the limited *SHL-Validation* set into vehicle- and user-specific sets. As a result, each of these sets contained insufficient data to train a robust model. Hence, we only proceeded with the vehicle-specific procedure in our final pipeline and tested the person clustering separately.

### 5.4. Feature Selection (2019 and 2020)

Since a large number of features was computed, the most relevant ones were selected using a three-step procedure. In the first step, the mutual information between each feature and the label was estimated, where a greater amount of mutual information implies a higher dependency between the feature and the label. In the second step, the Pearson correlation coefficient was computed for pairs of features. If the correlation was higher than the defined threshold, the feature with the lower mutual information with the label was discarded. In the final step, features were selected using a greedy-wrapper approach. A Random Forest classifier was first trained using only the best-scoring feature in the training set. The trained model was used to predict labels for the validation set and the prediction accuracy was calculated. Then, the second-best feature was added and the model was trained again. If the accuracy in the validation set was higher than that obtained without using this feature, said feature was retained. This procedure was repeated for all the remaining features. The reason for the first two steps is that the greedy-wrapper has a tendency to overfit to data when the number of features is large and the removal of correlated features reduces their number without this danger.

Feature selection is typically used to select the overall good features; however, in our case, we also used it to adapt the features to a particular phone location in 2019 and to particular users in 2020. The dataset that we adapted was used as the validation set during the feature selection procedure, with the intention that the process would select the features that transfer well form the training to validation set.

For 2019, we adapted the features to the *SHL-Validation* set, which only contains data from the *hand* location. The objective was to determine whether the features from different phone locations can be selected and adapted to the test location, which is not present in the training data. For this purpose, we explored three different adaptation schemes using different combinations of the training and validation data. In all four cases, the *SHL-Validation* set was split into quarters. The primary reason for creating quarters was to obtain a proper evaluation, i.e., one quarter was always left aside for testing, while the remaining three quarters were used for training and/or adaptation depending on the combination employed. The same adaptation procedure was performed for each quarter, and the final feature set was obtained as an intersection of all four selected feature sets.

For 2020, we adapted the features to the *SHL-Validation* set, which contains the data for User 2 and 3. For this purpose, we tested three different combinations of the training and validation data. Similar to the procedure used in 2019, in all three cases, the *SHL-Validation* set was divided into quarters. The same adaptation procedure was carried out for each quarter, and the final feature set was obtained as an intersection of all four selected feature sets.

We also selected features specific for the two clusters obtained through person clustering, to represent two different persons: Users 2 and 3. The *SHL-Validation* set was split into two clusters/users, as described in the previous section. Next, feature selection was carried out in the same manner as for User 2 and 3 combined: The two procedures were used on each cluster/user, and their union was taken as the final feature set for that user.

### 5.5. Temporal Smoothing Using an HMM (2019)

The SHL Challenge datasets are usually comprised of long-lasting activities, and fast transitions between different activities rarely occur. This means that, for example, situations in which the subway activity occurs once or twice within a longer sequence of cycling predictions are extremely unlikely, to the point where it is safe to say that the subway predictions are misclassifications. Further, it stands to reason that certain activity transitions are more likely to occur than others, which can contribute to the identification of missing activity predictions. One example of such a missing activity is when the user changes their mode of transportation without any detected walking activity in between.

One way of modeling and correcting such misclassifications is through the use of HMMs. HMMs learn rules (probabilities) from the labels of the data and the predictions that a classifier makes for these data. These rules can then be used to correct future predictions made by the same classifier.

When using HMMs, we use them in two slightly different capacities. The first, more straightforward application involves using them to smooth the predictions of a classifier in the time domain. The other involves providing alternative labels (knowledge) during the semi-supervised learning stage of the pipeline (described in Section 5.6). One of the uses of these alternative labels is to, for example, select an instance from the unlabeled data based on whether the classifier and HMM agree on what the label should be.

However, the use of an HMM is constrained by data that are temporally ordered. This condition was met after reordering the data from the 2019 SHL challenge but could not be met when using the data from the 2020 SHL Challenge. Thus, HMMs were excluded from the 2020 pipeline.

In the 2019 pipeline, when using HMMs to correct the predictions of a classifier for the test set, we used the *SHL-Train* set and the predictions (i.e. confusion matrix of an analogous classifier) to estimate the internal parameters of the HMMs. More specifically, we used the *SHL-Train* set to estimate the *transition_probabilities*, while we used the confusion matrix of the analogous classifier to estimate the *emission_probabilities*.

The analogous classifier is a classifier that is at the “same level/position” in a parallel pipeline that has all the same elements, except that instead of the *SHL-Train* set, *SHL-Validation* set, and *SHL-Test* set, it uses a four fold cross-validation schema for the *SHL-Train* and *SHL-Validation* sets to construct the training and test data. We assume that one can use its predictions to understand how to correct the main classifier’s predictions for the *SHL-Test* set.

### 5.6. Semi-Supervised Learning (2019 and 2020)

Both the 2019 and the 2020 SHL Challenge datasets used instances in the test set that were otherwise under-represented in the labeled data. In 2019, when constructing the *SHL-Test* set, the organizers used the data collected from the *hand* location: a category with extremely less representation in the labeled set that was released to the participants. In the 2020 *SHL-Test* set, they used data from two users who were only present in the *SHL-Validation* set, making this a significantly smaller labeled set than the *SHL-Train* set.

**Figure 2:**
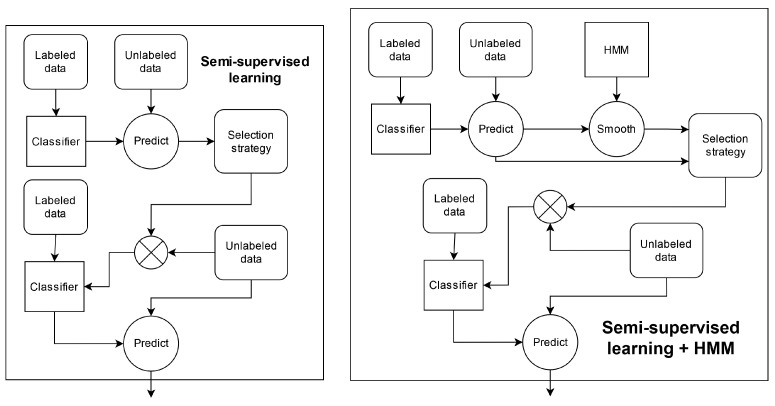
Diagrams for the general semi-supervised learning approach used in the *2020 pipeline* (**left**) and the semi-supervised learning approach that combines HMM predictions in the selection strategy (**right**). The second approach was used in the *2019 pipeline*.

To extract knowledge about these under-represented categories of instances from the *SHL-Test* set, semi-supervised learning was applied. Generally, semi-supervised learning is performed by (i) training a classifier on a set of labeled data; (ii) using the classifier to predict the labels of an unlabeled set of data; (iii) selecting instances from the unlabeled set with which to extend the labeled set using the labels generated in step (ii) and (iv) using the extended set of labeled data to train a new classifier. A diagram of the complete process can be seen in Figure 2.

A wide variety of procedures can be used to select instances from the unlabeled data in step (iii). For the *2020 pipeline*, we formulated the following selection strategy: use the instances for which the classifier gives a prediction probability that is above a certain threshold. We then investigated the effects of setting different threshold values between 0.1 and 1.0. The labels that were used for these instances after being selected were those assigned to them by the classifier.

In the case of the *2019 pipeline*, choosing a suitable selection strategy was slightly more complicated. In this pipeline, aside from the classifier predictions, we could also consider the version of the predictions that was post-processed using an HMM. Hence, we formulated several possible selection strategies: (i) use the instances for which the classifier provides a prediction probability that is above a certain threshold; (ii) use the instances for which the classifier provides a prediction probability that is above a certain threshold and for which the classifier and HMM agree with regard to the label; (iii) use the instances for which the classifier provides a prediction probability that is above a certain threshold and for which the classifier and HMM disagree with respect to the label; (iv) use the instances for which the classifier provides a prediction probability that is below a certain threshold and for which the classifier and HMM disagree with regard to the label; (v) use both the instances for which the classifier provides a prediction probability that is above a certain threshold and for which the classifier and HMM agree with regard to the label and those for which the classifier provides a prediction probability that is below a certain threshold and for which the classifier and HMM disagree with respect to the label. Furthermore, for each of these selection strategies, we investigated which labels should be used for the selected instances: the ones from the classifier or those from the HMM. Finally, for each combination of a selection strategy and label source, we tested several different threshold values.

The results of these experiments are given in Section 6.6.

### 5.7. Proposed Pipelines

**Figure 3:**
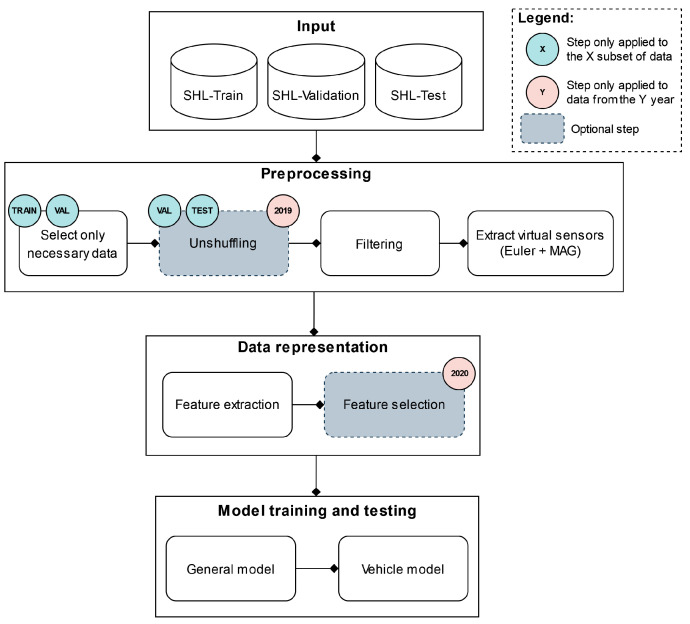
The general structure of the 2019 and 2020 pipelines.

Although the classification tasks presented in the 2019 and the 2020 SHL Challenges are fairly different, to address them, we propose two pipelines that share a large number of common characteristics. An overview of the general structure of both pipelines is shown in Figure 3. As is visible in Figure 3, up to the model training and testing stage, both pipelines follow a structure that is frequently used in the HAR domain. The input to each pipeline is the data provided by the organizers of that year’s challenge and consists of three subsets of data that have been segmented into five-second windows, as described in Section 3 and Section 4.3.

The next stage of both pipelines is the reprocessing stage. This stage consists of four different steps, of which one is optional and exists only in the 2019 pipeline. The first of these steps is data selection. As discussed in Section 6.2, training the classifiers using data from different sensor locations or different subjects yields very different performances; therefore, selecting the right combination of data is crucial.

The next step is unshuffling the *SHL-Validation* and *SHL-Test* sets to their original temporal order. This step is only applied to the data from the 2019 SHL Challenge and allows for the use of HMMs in the later stages of the classification pipeline. The unshuffling is carried out using the procedure described in Section 3.3. There is no need to perform this procedure on the *SHL-Train* set because its contents were not shuffled in the first place.

The final two steps in the preprocessing stage are filtering and extracting virtual sensor streams. Both steps are employed in both pipelines and are performed as described in Section 4.1 and Section 4.2.

The data representation stage of the pipelines is tasked with changing the way data are presented in later stages, i.e., it is tasked with extracting information from the raw data. This is conducted by performing feature extraction and, optionally, feature selection. During the feature extraction step, we calculated the extensive set of features described in Section 4.4. The feature selection step, on the other hand, exists only in the 2020 pipeline and was performed using the procedure described in Section 5.4.

Finally, the model training and testing stage consists of two major components: a general classifier and a so-called “vehicle” classifier. The role of the general classifier is to provide a label for all instances of the *SHL-Test* set, whereas that of the vehicle classifier is to reclassify the instances of the *SHL-Test* set that were not assigned a *run, walk, or bike* label by the general classifier. However, the implementation of this stage and, more specifically, the implementation of these high-level classifiers is where the proposed pipelines start to differ significantly. Thus, their inner workings will be explained separately in Section 5.7.1 and Section 5.7.2.

#### 5.7.1. Proposed Pipeline 2019

**Figure 4:**
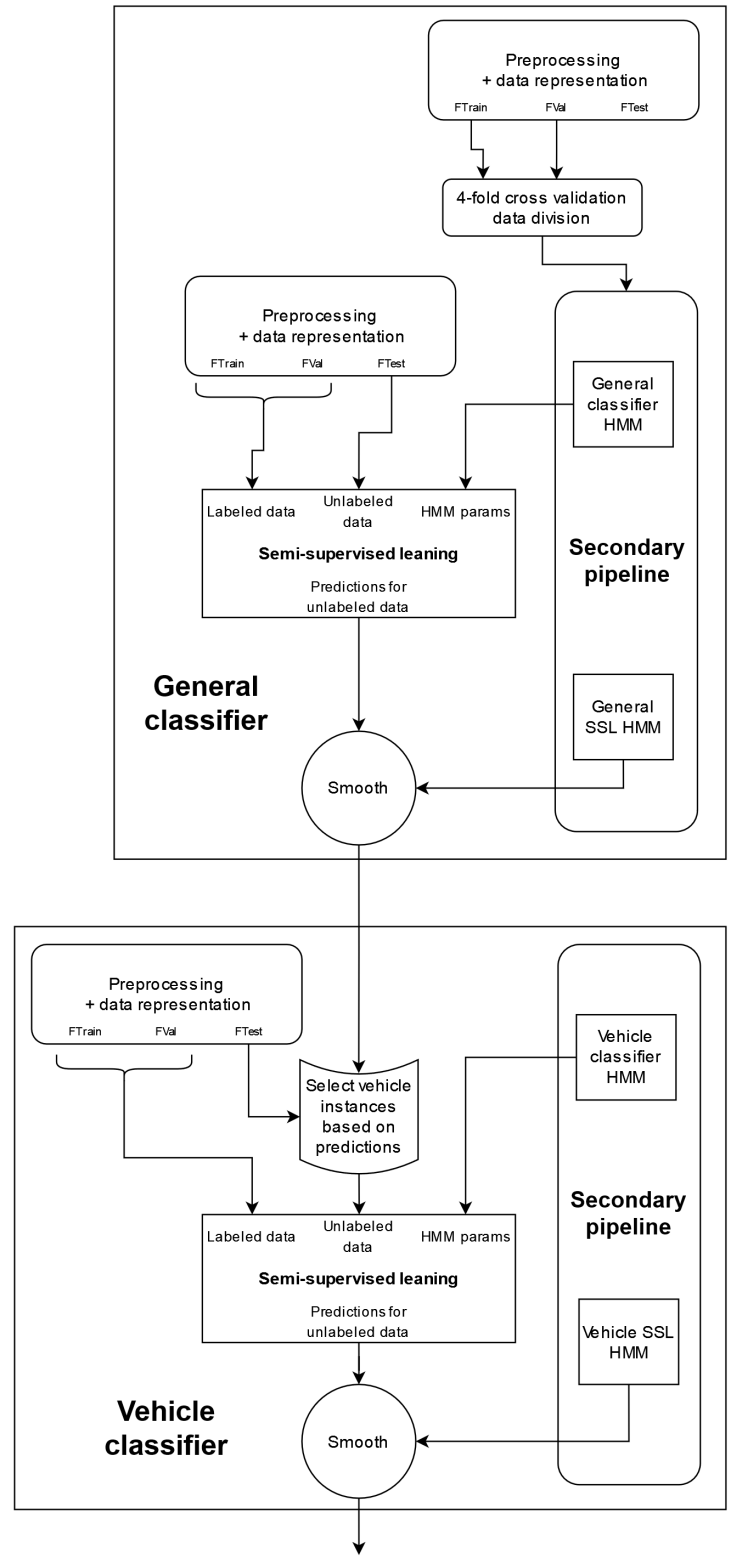
A diagram of the main classification pipeline used for the data from 2019.

**Figure 5:**
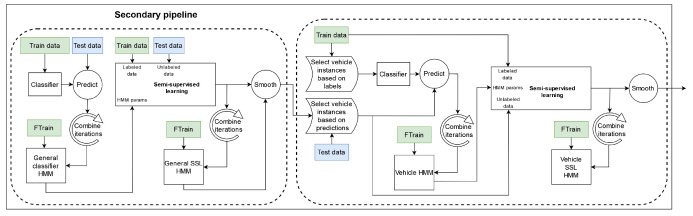
A diagram of the secondary (parallel) pipeline that provides the relevant HMM parameters.

The model training and testing stage of the 2019 proposed pipeline can be further divided into two parallel branches. The main part consists of the general and vehicle-specific classifiers and is concerned with predicting the labels of the instances in the *SHL-Test* set, while the other is a parallel pipeline that aims to generate relevant HMMs by performing four-fold cross-validation on the *SHL-Train* and *SHL-Validation* sets. The two parts intersect each time we use an HMM to post-process the predictions made by a certain classifier. Diagrams of both the main and the secondary pipeline can be seen in Figure 4 and Figure 5, respectively. It is important to note that the “*preprocessing + data representation*” block, shown in the figure, refers to the stages presented in Figure 3.

**Figure 6:**
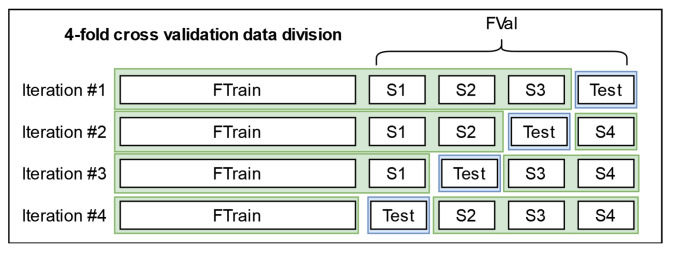
A four-fold cross-validation schema used for the internal testing of different pipelines using the 2019 or 2020 data.

The main pipeline begins by training a classifier using the semi-supervised learning method (with an HMM), as described in Section 5.6. The semi-supervised learning step uses the data from the *SHL-Train* and *SHL-Validation* sets as the labeled data and those from the preprocessed *SHL-Test* set as the unlabeled data. This semi-supervised learning step also utilizes an HMM (*general classifier HMM*), whose parameters are obtained in the secondary pipeline (shown in Figure 5). To determine the parameters of the HMM (*general classifier HMM*), in the secondary pipeline, we train a classifier and predict the labels for the instances of the test data four times: once during each iteration of the four-fold cross-validation depicted in Figure 6. We then combine the predictions made across the different iterations of the cross-validation schema and use them and the labels from the *SHL-Train* set to extract the parameters of an HMM (*general classifier HMM*), as described in Section 5.5.

After extracting the HMM parameters (*general classifier HMM*), for both the main and secondary pipelines, we start the process of semi-supervised learning. The selection strategy used in all the semi-supervised learning steps in this pipeline is explained in Section 6.6.

After the semi-supervised learning (SSL) procedure, we post-processed the predictions of the classifier again using an HMM (*general SSL HMM*). The parameters of this HMM were obtained in the same way as before, except that, this time, the predictions for the four test folds were generated using the (last) classifier trained during the semi-supervised learning step in the secondary pipeline. This step marks the end of the *general classifier* in both the main and secondary branches.

The function of the vehicle classifier is to re-predict the labels of those *SHL-Test* set instances that were not classified in the *walk, run, or bike* categories by the general classifier. This subset of instances was named the “*vehicle*” instances. To perform the reclassification, we divided the *SHL-Test* set into instances that need to be re-classified and those that do not based on the labels provided by the *general classifier*. From this point onward, the *test* set refers to the instances that require re-classification, i.e., the ones classified into a “*vehicle*” based activity. This notation, i.e., *train* and *validation*, extends to the *SHL-Train* and *SHL-Validation* sets, with the only difference being that, to divide the *SHL-Train* and *SHL-Validation* sets into “*vehicle*” and “*non-vehicle*” instances, we do not need to use the labels assigned by a classifier, as we can use the ground-truth labels. This selection of instances also occurs in the secondary branch, and everything described here applies to that as well.

Once we select only the “*vehicle*” instances from the *SHL-Train*, *SHL-Validation*, and *SHL-Test* sets, the steps to create a working vehicle classifier are exactly the same as those followed in the *general classifier* section of the pipeline. The *vehicle classifier* also uses semi-supervised learning (with an HMM) to train a classifier. This classifier is trained using the *train* data as the labeled set and the *test* data as the unlabeled set. Additionally, the HMM that is used to create this classifier is called the *vehicle classifier HMM*. The final step, as before, is to post-process the predictions of the classifier that were created during the semi-supervised learning procedure with a second HMM (*vehicle SSL HMM*). The parameters of the HMMs are extracted from the secondary pipeline in an analogous manner as before.

Once the *vehicle classifier* produces predictions, the final step of the pipeline is to combine the predictions of the *general classifier* with those from the *vehicle classifier*.

#### 5.7.2. Proposed Pipeline 2020

**Figure 7:**
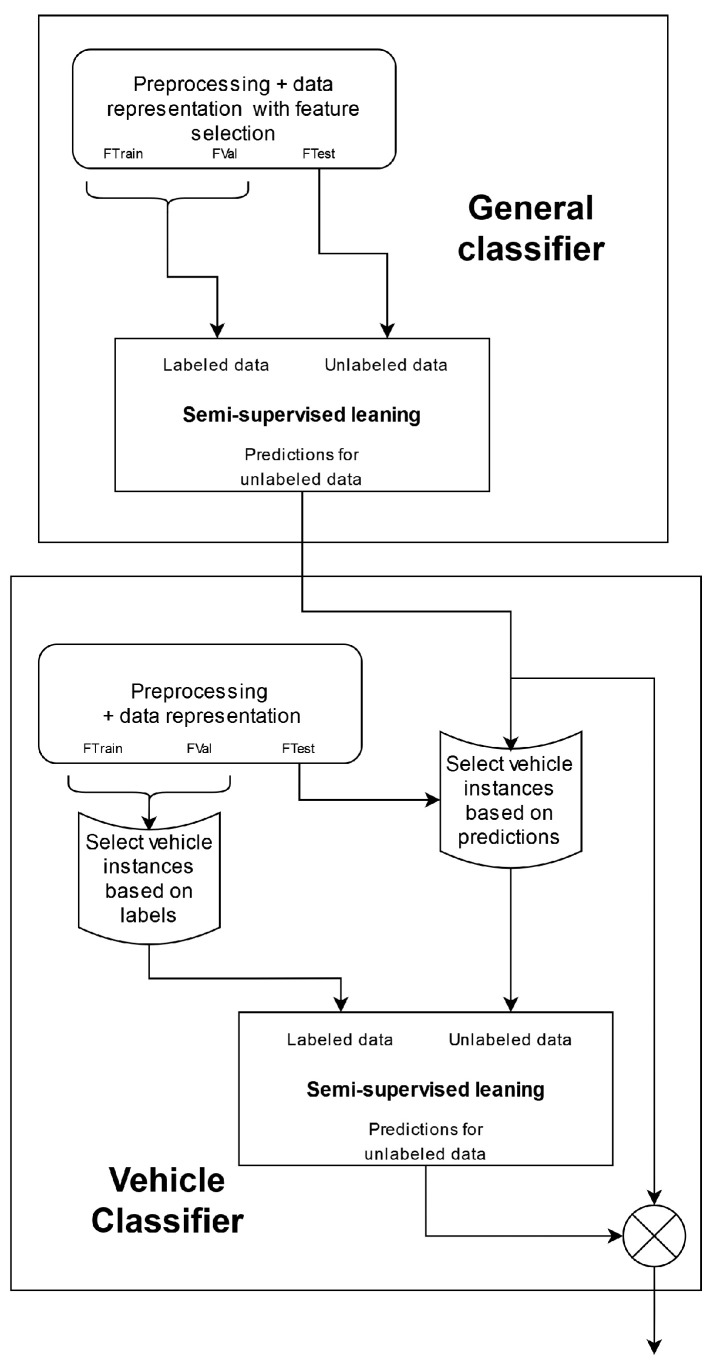
A diagram of the classification pipeline used for the data from 2020.

While the implementation of the model training and testing stage in the 2020 pipeline shares similarities with the 2019 one, it is made slightly simpler because the original temporal order of the instances in the 2020 *SHL-Test* set was not recoverable, which disallowed the use of HMMs. The exclusion of HMMs from this stage eliminated the need to extract the appropriate parameters for them and simplified the selection strategy of the semi-supervised learning method. However, it also made the method slightly less effective. The implementation of the *general classifier* and the *vehicle classifier* in the 2020 pipeline is shown in Figure 7.

It is important to note that although feature selection is performed in this pipeline, only the *general classifier* uses the output of the feature selection step, while the *vehicle classifier* does not, and instead uses the full feature set. This is emphasized in Figure 7 by adding the “with feature selection” text to the *preprocessing and data representation block* (which refers to the stages shown in Figure 3).

The *general classifier* in this pipeline serves the same function as the one in the 2019 pipeline. However, this *general classifier* is less complex, as it was trained using the semi-supervised learning procedure that does not include the use of an HMM (presented in Section 5.6).

After the *general classifier*, the data in the *SHL-Test* set are split into two subsets: a “*vehicle*” subset and a “*non-vehicle*” subset. These subsets retain the same meaning as those mentioned in Section 5.7.1. We then continue to re-classify the instances of the “*vehicle*” subset using a classifier that was trained using the semi-supervised learning methodology.

## 6. Experimental Results

### 6.1. Recognition of Unknown Location (2020)

To evaluate the performance of the phone location detection, a four-fold cross-validation technique was utilized. Each fold represented exactly one quarter of the provided *SHL-Validation* set. Although the validation set contains a few data samples, it provides more relevant information about the phone position in the test set, as it is comprises data recorded from the same user as the test user.

The first step toward the detection of the phone location involved the recognition of the dynamic activities, i.e., walking or running (see Section 5.1). The walking/running recognition showed an F1 score of 0.93. However, the overall score achieved during this step was not a primary concern for the purpose of phone location detection. Instead, we were more interested in the percentage of false positives (i.e., the percentage of instances that were incorrectly classified as walking/running) because they could have a large impact on the performance of the phone location classifier. Nevertheless, only 2% of all instances from the validation set were incorrectly classified as walking/running activity, and given that 88% of the instances labeled as walking/running activity were true positives, no further improvements were needed. The results from the walking/running activity recognition for the validation set are presented in Table 2.

In the second step, the phone location was identified based solely on the instances that were classified as walking/running in the previous step. This implies that the true positives and the false positives were included here. In this experiment, the highest F1 score was noted for the *torso* location (0.86). The F1 scores for the remaining locations were as follows: 0.84 for the *bag*, 0.81 for the *hand*, and 0.80 for the *hips*. The results from the phone location detection in the validation set are presented in Table 3.

These results are probably adequate for ensuring that high classification accuracy is attainable using phone detection. Therefore, the phone location detection for the *SHL-Test* set was carried out in the next step. The entire *SHL-Validation* set was used to train a final model for the detection of walking/running activity, which was then used to classify the instances from the *SHL-Test* set. A total of 15,464 instances from the *SHL-Test* set were classified as walking/running activity. Subsequently, 83.4% were classified as *hips* location, 15.7% were classified as *hand* location, while fewer than 1% were classified as either *bag* or *torso* location. Based on these results, we concluded that the test set’s unknown phone location is *hips*. The results for the test set related to location detection are presented in Table 4.

### 6.2. Impact of Training Data and Feature Selection for 2019 and 2020

Since the 2019 and 2020 SHL datasets consist of a large training set that is somewhat different from the test set, and a validation set that is smaller but more similar to the test set, this section focuses on the best ways of combining these sets for training. Additionally, for each training data combination, we explored whether feature selection can help adapt the models to the particular test location (2019) or person (2020).

For both 2019 and 2020, a four-fold cross-validation was performed on the *SHL-Validation* set, where each step was one-fourth of the validation set. All the results for 2019 were obtained using instances from the *hand* location, as it was the only one provided in the *SHL-Test* set. Similarly, for 2020, the results were obtained using instances from the *hips* phone.

For 2019, the aim of the experiment was to determine whether one should use the *SHL-Train* set for training because it does not include any instances from the phone location present in the test data (*hand*). Furthermore, this experiment was also conducted to determine whether a combination of the *SHL-Train* and *SHL-Validation* (where we have some *hand* data) sets could yield higher classification accuracy. For 2019, we tested four combinations. The first used only the *SHL-Train* set for training, while the second used the *SHL-Train* set and three-fourths of the *SHL-Validation* set. For the third combination, we used the *SHL-Train* set, and the data for only the *hand* location from the *SHL-Validation* set. The last combination was used only for the *SHL-Validation* set.

The results obtained for 2019 are shown in Table 5. The columns of this table show which phone location was used for training. *All* refers to all phone locations except *hand*. The first combination shows that the best results were obtained when the model was trained using the data from all phone locations present in the *SHL-Train* set (*bag*, *hips*, *torso*). Additionally, it can be seen that when the features are adjusted to the *hand* location using feature selection, the results are significantly improved for all training locations. The same behaviour was observed for the second combination regardless of the additional data included in the validation. This was expected because only another three days of data were added to the training dataset. However, for the third combination, where we used *hand* data from the *SHL-Validation* set together with the other locations from the *SHL-Train* set, a significant improvement is shown. Unlike the previous two combinations, the feature adaptations using the feature selection procedure did not produce any improvement. This could be expected because the training data comprised instances from multiple phone locations and because the feature selection procedure cannot select the appropriate features that work for multiple locations concurrently. The results demonstrated that the *SHL-Train* data from the *hips* location, in combination with the *SHL-Validation* data from the *hand* location produced the best performance. Finally, the last combination, where data from only the *SHL-Validation* set were used, illustrated the performance of a location-specific model. This model achieved an F1 score of 0.71 when trained on approximately three days of data. However, our experiment demonstrated that it could be beneficial to combine data from multiple locations when insufficient data from the target location are available. Furthermore, the improvement introduced using the *hips* location in combination with *hand* location can be expected because, in most of the activities, the leg is performing the same repetitive movement as the *hand*.

For 2020, once the phone location of the test data was determined, we explored whether a location-dependent model would yield a better performance than a general location-independent model. Moreover, the main idea behind testing different combinations of data from the *SHL-Train* and *SHL-Validation* sets was to verify whether using data (*SHL-Train*) from a subject who is not present in the *SHL-Validation* and *SHL-Test* sets could be beneficial.

Three different combinations of training data were tested. The first used only the *SHL-Train* set for training, while the second used the *SHL-Train* set and three-fourths of the *SHL-Validation* set. The last combination used only the *SHL-Validation* set. Unlike the 2019 data, here, the target location (*hips*) appears in both the *SHL-Train* and *SHL-Validation* sets.

The results obtained for 2020 are shown in Table 6. The columns of this table indicate which phone location was used for training. Here, *All* refers to the data from all phone locations, namely *bag*, *torso*, *hand* and *hips*. The combinations that included the *hips* variable were not tested with the rest of the locations separately, as the 2019 results showed that when sufficient training data of the target location are available, it is best to train the model using either the target location alone or using all training locations. The first combination demonstrated that training a location-specific model results in a higher F1 score. However, the F1 score is relatively low, which suggests that there is a big difference between the data of User 1 from the *SHL-Train* set and those of User 2 and 3 from the *SHL-Validation* set. Therefore, the features extracted with data from User 1 were also adjusted to User 2 and 3, which provided significant improvements. In addition, the results indicate that using the data from all phone locations in combination with the feature selection step yields a small improvement compared to a location-specific model. The second combination, where the data from the *SHL-Train* and the *SHL-Validation* sets are combined, resulted in another large improvement compared to the previous combination. This confirms the assumption that User 1’s data are considerably different from those of User 2 and 3, and indicates that the best solutions are often domain dependent. The second combination also provides a small improvement over the location-specific model when feature selection is used. Finally, the results of the last combination, where only User 2’s and 3’s data were considered, are worse than those of the second combination. Based on these findings, we can conclude that when only a small portion of data from the target user are available, better results can be achieved by utilizing data from different users and training a more robust model.

### 6.3. Selection of the Machine-Learning Algorithm for 2019 and 2020

After choosing the best combination of training data and selecting the appropriate feature set for each year, different ML algorithms were tested. Along with the F1 scores, the training time of the algorithms was also noted. The results obtained from this experiment for both 2019 and 2020 are shown in Table 7. All models were trained with the default scikit-learn hyperparameters. The results shown in the table were obtained using the *SHL-Validation* set.

For 2019, it can be seen that all models, except a decision tree, perform similarly. The highest F1 score of 0.71 was obtained using Support Vector Classification (SVC). However, when also considering the training time for each model, the performance of SVC is the second worst. Therefore, we opted for the Random Forest algorithm, mainly because it provides stable results and has the shortest training time. Additionally, it does not require much parameter tuning.

The behavior of the 2020 trained models was similar. Again, the results obtained using a decision tree were the worst, although the difference is smaller. Further the best results were obtained with a Random Forest model. The same F1 score of 0.82 was also obtained with an AdaBoost classifier that used Random Forest as the base classifier. However, since it did not offer any improvement over Random Forest, the latter remained the primary choice.

### 6.4. Vehicle Models 2019 and 2020

To evaluate the performance of the vehicle- and non-vehicle-specific models, we used the experimental setup described in Section 6.2. Once again, for both vehicle and non-vehicle models, we tested which combination of data from the *SHL-Train* and *SHL-Validation* sets results in the most accurate recognition of activities. Furthermore, various combinations of data from different phone locations were also tested.

First, we assumed that there an adequate binary classifier exists, which can distinguish between vehicle-specific transportation modes (being stationary, car, bus, train, and subway) and non-vehicle transportation modes (walking, running, and biking). Next, vehicle- and non-vehicle-specific models were trained for each combination of training data. Finally, predictions were generated for the validation data. The data utilized for this were divided into vehicle- and non-vehicle-specific transportation modes based on the ground truth. This entails that the obtained results provide information about the performance of the models if information regarding which instance belongs to the vehicle or non-vehicle transportation modes is available. Since this is not the case in practice, and we do not know whether a specific activity is vehicle or non-vehicle related, these results should be considered a comparison between the combinations.

For 2019, the results for the vehicle-specific model demonstrate that the best performance is obtained when training on the *hips* location from the *SHL-Train* set and the *hand* location from the *SHL-Validation* set. For the non-vehicle model, the results were the same for two combinations: training on the *hips* location from the *SHL-Train* set and the *hand* location from the *SHL-Validation* set and training using only the *hand* location from the *SHL-Validation* set. The former was chosen because it is the same combination as that used for the non-vehicle model, thus reducing the implementation complexity of the pipeline.

For 2020, the best combination for both vehicle- and non-vehicle-specific models involves using the *hips* location trained with data from the *SHL-Train* and *SHL-Validation* sets.

Finally, for both years, we tested whether the vehicle- and non-vehicle-specific models produce any improvement over a general classifier, trained using all the classes together. These results are displayed in Table 8. All the results associated with vehicle- and non-vehicle specific models were obtained using instances that truly belonged to either of these two classes. To verify whether these models yielded any improvement, we used them in addition to our general classifier. To assess whether both the vehicle- and the non-vehicle models are useful, we conducted a step-by-step evaluation. First we evaluated the performance using only reclassification performed with the vehicle model. Next, we reclassified the predictions of the general model using only the non-vehicle model, and, finally, we used both the vehicle- and the non-vehicle model together. Based on the results obtained for both 2019 and 2020, the same outcome was achieved. The vehicle-specific model was relatively superior. However, the non-vehicle models did not affect the performance of the general classifier. Furthermore, the combination of both vehicle and non-vehicle models demonstrated that the non-vehicle model is not useful in this particular situation. Therefore, we decided to proceed with the combination that uses the general classifier along with the vehicle-specific classifier.

### 6.5. Person Clustering

**Figure 8:**
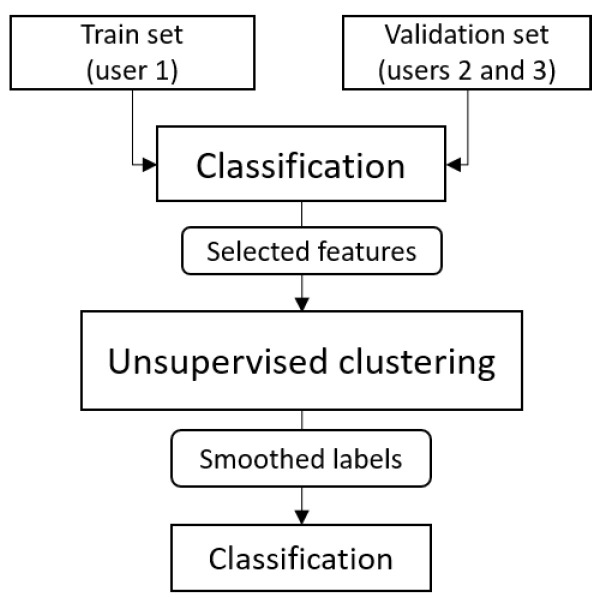
Clustering pipline.

**Figure 9:**
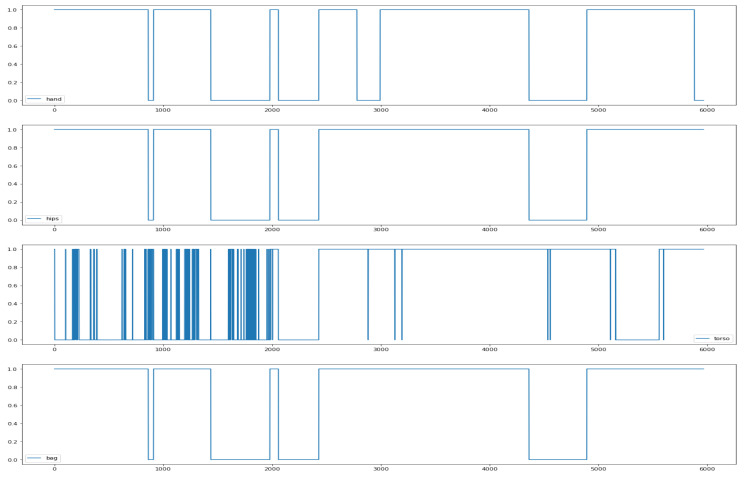
Example of clustered data for all four locations (from top to bottom: *hand*, *hips*, *torso*, and *bag*) for walking. The clustering is applied in the same manner as that for all other activities.

We performed person clustering, as described in Section 5.3 and shown in Figure 8. The classification problem of separating the *SHL-Train* set from the *SHL-Validation* set was addressed using the Random Forest classifier. Fifty of the most important features for each location according to Random Forest’s internal feature importance measure were selected to cluster the *SHL-Validation* set. Two clusters were obtained (silhouette score 0.58). To ensure that the clustering did not just cluster the activities, the clustering algorithms were run on each of the activity subsets and compared with the clusters obtained when running the clustering algorithm on the entire *SHL-Validation* set. It was found that the clusters matched. For three locations (*hand*, *hips*, and *bag*), the clusters were very similar, as can be seen in Figure 9. Clustering did not work as well for the *torso* location.

We then smoothed the clusters by ensuring that all of the consecutive samples were in the same cluster. The smoothed clusters were used to build a Random Forest classifier that predicted the clusters for the *SHL-Test* data.

Once we defined the different clusters, the next step was to train cluster-specific models. For this purpose, both clusters were divided into quarters. For each cluster, we trained models using the *hips* data from the *SHL-Train* set and three-fourths of the cluster data from the *SHL-Validation* set. In this way, we generated predictions for all quarters for both clusters. Finally, all the predictions were combined, and an F1 score of 0.84 was obtained for the *SHL-Validation* set. We also tested the performance of the cluster-specific approach for the *SHL-Test* data and obtained an F1 score of 0.785. This suggested that the cluster-specific approach was slightly overfitted to the validation data.

### 6.6. HMMs and Semi-Supervised Learning

After establishing that the use of vehicle-specific models was beneficial based on the findings discussed in Section 6.4, the next step was to investigate whether using HMMs, semi-supervised learning, or a combination of the two could improve the results.

**Figure 10:**
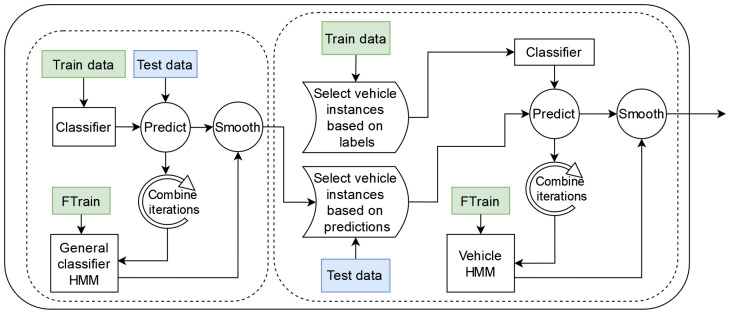
The pipeline used to test the usefulness of HMMs on the data from 2019.

We began by testing the usefulness of HMMs when working with the data from 2019 using a similar pipeline to the four-fold cross-validation (secondary) pipeline presented in Section 5.7.1. Although similar, this pipeline is simpler because it does not include any semi-supervised learning elements. The pipeline can be seen in Figure 10.

To reiterate, the pipeline was executed four times (concurrently), as that is the number of folds in our cross-validation scheme. The predictions of concurrent executions are gathered at two points in the pipeline, which is when they are used to produce an accurate confusion matrix. These confusion matrices and the labels of the *SHL-Train* set were used to estimate the internal parameters of two different HMMs, which are depicted as *general classifier HMM* and *vehicle HMM*. These HMMs were then used to post-process the predictions of the *general classifier* and *vehicle classifier*.

The results of these experiments are shown in Table 9. They demonstrate that using HMMs distinctly enhances the performance of regular classifiers. However, using HMMs to post-process the predictions of both the *general classifier* and *vehicle classifier* seems to diminish the improvements brought about by using a vehicle-specific classifier. This is illustrated by the small difference between the F1 scores presented in the second and last row of Table 9.

After confirming the hypothesis that using HMMs is beneficial when working with data from the 2019 Challenge, we started testing whether the inclusion of semi-supervised learning would yield similar improvements. This led to the question of which predictions should be used when selecting instances for the semi-supervised learning method. It could be valid to consider not only the predictions of the classifier during the selection process but also the post-processed predictions. Several different selection strategies were tested for the semi-supervised learning method. More specifically, we tested one strategy that did not use the post-processed predictions and eight strategies that used different ways of combining those two types of predictions to select suitable instances. During these experiments, we used the same four-fold cross-validation (secondary) pipeline presented in Section 5.7.1. The only aspect that changed in each experiment was the selection strategy employed. The obtained results are directly comparable in the last row of Table 9.

The findings from the aforementioned experiments are presented in Table 10. Each row of the table presents the results of a different selection strategy. The first column displays the conditions that constitute a specific selection strategy, while the second column indicates which predictions we take as ground-truth labels for our selected instances. Columns 3–7 show the results for each selection strategy when using different thresholds (abbreviated as *thr* in the selection strategy cells). The last column indicates the optimum results that a selection strategy was able to achieve across all thresholds.

The first row of the table presents the results of a selection strategy that does not consider the post-processed labels and makes its decisions based only on the predictions from the classifier. The other rows of the table present selection strategies that account for both the classifier and the post-processed predictions. From the presented results, one can observe that combining both types of predictions in the selection strategy almost always produces better results than when using only the predictions made by the classifier. Furthermore, the best results are produced when one selects those instances of the *SHL-Test* set for which the classifier is relatively unsure and whose label the classifier and HMM disagree on. In this case, our experiments suggest that it is best to use the labels that the classifier provides as the ground truth.

In contrast, working with the data from the 2020 SHL Challenge was less complex. Since the *SHL-Test* data could not be temporally sorted, it was not possible to utilize HMMs. Nonetheless, semi-supervised learning could be employed to train both the *general classifier* and the *vehicle classifier*. To test the effectiveness of semi-supervised learning, we used a pipeline that is nearly identical to the one shown in Figure 7.

The pipeline consists of two classifiers, a *general classifier* and a *vehicle classifier*, that were trained using semi-supervised learning. The only difference between this pipeline and the one displayed in Figure 7 is the fact that this one uses the *SHL-Train* and three-fourths of the *SHL-Validation* set as the labeled data and the last quarter of the *SHL-Validation* set as the unlabeled test data (splits in the data are created by a four-fold cross-validation scheme such as the one presented in Figure 6). The results of this pipeline are shown in Table 11 and are directly comparable to those presented in Section 6.8.

Based on the results of our experiments, it is clear that using semi-supervised learning is beneficial, as it improves the previous results by several percentage points when using the data from both the 2019 and the 2020 SHL Challenge. The results also highlight the usefulness of HMMs and how effectively they can be combined with a semi-supervised learning methodology.

### 6.7. Proposed Pipeline 2019

Since the pipeline and each of its elements have been described in Section 5.7.1, we can analyze its performance at several stages.

To estimate the performance of the proposed pipeline using only the *SHL-Validation* set, we employed a four-fold cross-validation scheme and the pipeline depicted in Figure 5. Further, when calculating the performance of the proposed pipeline for the *SHL-Test* set, one could use the entirety of the *SHL-Train* and *SHL-Validation* sets to train the models and extract the HMM parameters. The pipeline that produces results on the test set is shown in Figure 4. A summary of the performance (of the different stages) of the proposed pipeline for the *SHL-Validation* set and *SHL-Test* sets is provided side-by-side in Table 12.

Our pipeline slightly overfits for the *SHL-Train* and *SHL-Validation* sets, which results in a four percentage point difference between the final results on the test set and those obtained using four-fold cross-validation. Additionally, it seems that the performance boost during the four-fold cross-validation evaluation, using semi-supervised learning to train the *General classifier* is negligible—as observed from the almost identical *SHL-Test* scores in rows two and three. Using semi-supervised learning seems to overfit the classifier, thus producing a slightly lower score. There is, however, a 1.5 percentage point increase when using semi-supervised learning to train the *Vehicle classifier*. This is indicated by the increase in *SHL-Test* performance between rows four and five. Finally, the performance increase observed when using Hidden Markov Models seems to be consistent when evaluating on the test set.

It is important to note that our first-place result on the 2019 SHL Challenge was 0.7842, which means that this new pipeline improved the score by about 2.5 percentage points. This is also an estimation of the top results obtained by the existing methodology.

### 6.8. Proposed Pipeline 2020

The internal estimation of the performance of the pipeline was carried out using a four-fold cross-validation scheme, such as the one depicted in Figure 6. Since the use of HMMs is not possible for these data, the pipeline is less complicated than that proposed for the 2019 data, although it retains most of the components. The pipeline used for the evaluation on the *SHL-Validation* set is identical to that used for the *SHL-Test* set (shown in Figure 7), with the exception that it uses the training and test data provided by the four-fold cross-validation scheme as the labeled and unlabeled data, respectively.

A summary of the results can be found in Table 13. Again, the pipeline seems to slightly overfit on the *SHL-Train* and *SHL-Validation* sets, as represented by the difference between the final scores for these sets (0.846 and 0.8, respectively). Additionally, the usefulness of the *vehicle classifier* seems to be overestimated by the cross-validation scheme, as its impact on the results of the test set is quite small.

Finally, this pipeline also yielded an improvement in the results as compared to those achieved in the 2020 SHL Challenge. The improvement was around 2.1 percentage points.

## 7. Conclusions

In this paper, we use the 2019 and 2020 Sussex-Huawei Activity Recognition Challenges as a framework to analyze the usefulness of commonly used activity recognition pipeline components in situations with a significant domain shift. Specifically, this paper expands on our previous competition entries (placed first and third in the 2019 and 2020 challenges, respectively), to analyze how well different ML methods generalize across recognition tasks and how much they actually contribute to the overall classification performance.

Following the flow of a relatively standard activity recognition pipeline, we will first present our conclusions regarding the choice of training data, feature extraction and the use of feature selection techniques, in that order.

In terms of selecting the appropriate training data, our experiments show that supplementing a small training set with data that do not perfectly match the distribution of the test set can still be very helpful. This is shown by the fact that, in both the 2019 and 2020 SHL Challenges, using a combination of the *SHL-Train* and *SHL-Validation* sets performs better than using only data from the *SHL-Validation* set, even though the *SHL-Train* set exhibits a significant domain shift in comparison to the *SHL-Test* set. However, it also seems that extending the training data indefinitely does not always result in an increase in the observed performance and there exists a tipping point at which the training data becomes sufficiently large such that adding more suboptimal data results in a decrease in performance. This can be seen by the fact, that in both the 2019 and 2020 SHL Challenges, adding data from the *SHL-Train* set from sensors in locations that are not compatible with the senor location in the *SHL-Test* set ends up decreasing the performance of the pipeline.

Furthermore, since our experiments showed that aligning the training and testing data as best as possible is crucial, it is important to note the results of our experiments aiming to detect sensor placement (location) as well as those aiming to cluster the data into subsets that would represent different people. Namely, we found that detecting the placement (location) of a sensor is possible, relatively straightforward and moderately beneficial. Detecting and using the appropriate training data for the sensor location in the *SHL-Test* set, brought an improvement of around 1 p. p. On the other hand, detecting which data belong to which user was a more difficult task and appeared to be less helpful.

In terms of feature extraction and selection, we came to the conclusion that beginning one’s analysis using a big set of relevant features, such as the ones extracted by our feature extraction library [55], is beneficial and, in some cases, reducing the number of those features using feature selection could also help to increase the overall performance of the pipeline. Unfortunately, based on our results, the usefulness of feature selection is inconclusive and should be explored on a case-by-case basis.

Continuing down the components of a standard activity recognition pipeline, we next present our conclusions about the choice of the ML algorithm, the use of different models for different activities and the use of the semi-supervised learning paradigm.

In terms of choosing an appropriate ML algorithm, our experiments show that using a Random Forest Classifier is a solid choice. In fact, based on our results here and in our previous work [5], using ensemble algorithms seems to be a good idea in general. Additionally, in this study, using a Support Vector Classifier also showed a good performance, even though this is not usually the case.

When it comes to training models for specific activities (e.g., a vehicle classifier) or users and creating a hierarchy of such models, our experiments indicate moderate performance benefits. The same is true when using semi-supervised learning to train classifiers.

Finally, regarding the use of HMMs to post-process the predictions of a classifier, our experiments show that, in situations where their use is possible, they provide significant performance benefits of up to 10 p. p. In fact, since they are not too complex to implement, we recommend using them if the activities to be recognized are not too short and their temporal relation has some degree of regularity.

Although we have already considered two slightly different datasets and tested the usefulness of pipeline components in two different scenarios, confirming our conclusions in a larger set of scenarios would be a significant contribution. This is why, in future, we plan on extending this analysis using a greater number of diverse activity recognition datasets. This is especially important to further explore the impacts of methods that we could only apply to one of the challenges, such as person clustering and the combination of semi-supervised learning and HMMs.

## Figures and Tables

**Table 1:** Summary of the datasets provided for 2019 and 2020. “X” denotes whether data from that location were present in the subset and whether the subset was labeled.

2019						
	**Bag**	**Torso**	**Hips**	**Hand**	**Labels**	**Days**
*SHL-Train*	X	X	X		X	59
*SHL-Validation*	X	X	X	X	X	3
*SHL-Test*				X		20
**2020**						
	**User 1**	**Users 2 & 3**		**Locations**	**Labels**	**Days**
*SHL-Train*	X			4	X	59
*SHL-Validation*		X		4	X	6
*SHL-Test*		X		1 (unknown)		40

**Table 2:** Activity recognition confusion matrix.

	Predicted	Walking/Running	Other
True			
Walking/Running		20,391	2729
Other		2532	92,233

**Table 3:** Location detection confusion matrix.

	Predicted	Bag	Hand	Hips	Torso
True					
Bag		4050	378	558	334
Hand		24	5207	19	48
Hips		9	1529	5240	280
Torso		222	387	218	4420

**Table 4:** The number of instances from each phone location in the test set, as predicted by our location detection model.

	Bag	Hand	Hips	Torso
No. of instances	95	2430	12,901	38

**Table 5:** Comparison of the F1 scores obtained using different combinations of training data for 2019. For each training combination, we tested whether the adaptation of the features to the *hand* location improves the results. All the results listed in this table were obtained using only instances from the *hand* location when testing. For the last combination, where data only from the *SHL-Validation* set were used, * denotes that no instances from *hand* location were used for training.

	FS	All	Bag	Hips	Torso	Hand
Train: *SHL-Train*, Valid: *SHL-Validation*	√	0.53	0.51	0.36	0.48	X
	√	0.67	0.59	0.5	0.61	X
Train: *SHL-Train* + 3/4 *SHL-Validation*, Valid: 1/4 *SHL-Validation*	√	0.53	0.515	0.36	0.475	X
	√	0.62	0.58	0.43	0.56	X
Train: *SHL-Train* + 3/4 *SHL-Validation* (hand), Valid: *SHL-Validation*	√	0.755	0.754	**0.765**	0.735	X
	√	0.74	0.724	0.73	0.68	X
Train: 3/4 *SHL-Validation*, Valid: 1/4 *SHL-Validation*	√	0.41 *	0.27	0.25	0.39	0.71
	√	0.52 *	0.29	0.25	0.46	0.69

**Table 6:** Comparison of the F1 scores obtained using different combinations of training data for 2020. For each training combination, we tested whether the adaptation of the features to the *hips* location improves the results. The columns *All* and *hips* indicate which data were used for training. The *All* column includes *bag*, *torso*, *hand* and *hips*. All the results presented in this table were obtained using only the instances from the *hips* location when testing.

		All	Hips
Trained on *SHL-Train*, validated on *SHL-Validation*	Without FS	0.56	0.6
	With FS	0.71	0.7
Trained on *SHL-Train* + 3/4 *SHL-Validation*, validated on 1/4 *SHL-Validation*	Without FS	0.81	0.81
	With FS	0.81	**0.82**
Trained on 3/4 *SHL-Validation*, validated on 1/4 *SHL-Validation*	Without FS	0.79	0.78
	With FS	0.76	0.72

**Table 7:** Comparison of the F1 scores and training times obtained for different classical ML methods for 2019 and 2020.

Method	2019		2020	
	F1 Score	Training Time (s)	F1 Score	Training Time (s)
XGBoost	0.69	2644	0.78	2923
Decision tree	0.57	427	0.71	612
Random forest	0.70	1390	0.82	1566
Gradient boosting	0.70	20,341	0.79	22,415
Bagging	0.66	7468	0.78	8153
SVC	0.71	17,688	0.77	19,353
AdaBoost with RF	0.70	1827	0.82	1964

**Table 8:** The F1 scores obtained when the vehicle and non-vehicle models are combined with the general classifier. This table illustrates whether the vehicle and non-vehicle models introduce any improvement over the general classifier.

	2019	2020
	F1 Score	F1 Score
General classifier	0.696	0.82
General classifier + vehicle model	0.71	0.834
General classifier + non-vehicle model	0.696	0.82
General classifier + vehicle model + non-vehicle model	0.71	0.834

**Table 9:** The F1 scores obtained at different stages of a pipeline that uses a vehicle-specific model and HMMs. The pipeline utilizes data from the 2019 SHL Challenge.

Pipeline	F1 Score
General classifier	0.696
General classifier + HMM	0.83
General classifier + HMM + vehicle classifier	0.731
General classifier + HMM + vehicle classifier + HMM	0.832

**Table 10:** The F1 scores produced by different semi-supervised learning selection strategies when working on the data from the 2019 SHL Challenge.

Selection Strategy	Label	0.1	0.3	0.5	0.7	0.9	Best
clf_proba > thr	CLF	83.18	82.62	81.63	83.16	83.27	83.27
clf_proba > thr	HMM	83.18	83.31	82.62	83.5	83.66	83.66
clf_proba textgreater thr; CLF & HMM agree	CLF	82.23	82.76	82.73	83.27	83.1	83.27
clf_proba > thr; CLF & HMM disagree	HMM	83.44	83.37	83.43	82.83	83.66	83.66
	CLF	83.79	79.67	83.11	82.68	82.97	83.79
**clf_proba < thr;** **CLF & HMM disagree**	HMM	83.49	83.44	83.53	83.22	83.18	83.53
	**CLF**	**83.02**	**80.05**	**79.98**	**84.98**	**83.18**	**84.98**
clf_proba > thr; HMM & CLF agree or clf_proba < thr; HMM & CLF disagree	HMM	82.23	83.17	83.39	83.37	83.65	83.65
	CLF	82.23	82.29	82.35	82.48	80.29	82.48

**Table 11:** The F1 scores produced by a pipeline that uses classifiers trained with semi-supervised learning when working on the data from the 2020 SHL Challenge.

Selection Strategy	0.1	0.3	0.5	0.7	0.9	Best
clf_proba > thr	83.60	83.75	**84.64**	83.82	83.58	84.64

**Table 12:** A comparison of the F1 scores produced by different stages of the pipeline using a four-fold cross-validation scheme on the *SHL-Validation* set and on the official *SHL-Test* data. Abbreviations used in the table: GC—General classifier, HMM—Hidden Markov Model, SSL (X, HMM)—semi-supervised learning for classifier X, which uses the labels produced by the classifier X and an HMM tuned to that classifier to select instances for further training, VC—vehicle classifier.

Stage of the Pipeline	Validation F1 Score	Test F1 Score
GC	0.69656	0.7113
GC + HMM	0.83007	0.8078
SSL (GC, HMM) + HMM	0.83803	0.8037
SSL (GC, HMM) + HMM + VC + HMM	0.83146	0.7938
**SSL (GC, HMM) + HMM ** **+ ** **SSL(VC, HMM) + HMM**	**0.8498**	**0.809**

**Table 13:** A comparison of the F1 scores produced at different stages of the 2020 pipeline using a four-fold cross-validation scheme for the *SHL-Validation* set and the official *SHL-Test* data. The abbreviations used in the table are: GC—General classifier, SSL (X)—classifier X trained using semi-supervised learning, VC—vehicle classifier.

Stage of the Pipeline	Validation F1 Score	Test F1 Score
GC	0.794	0.783
SSL (GC)	0.798	0.791
SSL (GC) + VC	0.837	0.7953
SSL (GC) + SSL (VC)	0.846	0.8

## Data Availability

The dataset used in this study can be found at: http://www.shl-dataset.org/ (accessed on 1 May 2022).

## References

[1] Wang L., Gjoreski H., Ciliberto M., Mekki S., Valentin S., Roggen D. (2019). Enabling Reproducible Research in Sensor-Based Transportation Mode Recognition with the Sussex-Huawei Dataset. IEEE Access.

[2] Wang L., Gjoreski H., Ciliberto M., Mekki S., Valentin S., Roggen D. (2018). Benchmarking the SHL Recognition Challenge with Classical and Deep-Learning Pipelines. Proceedings of the 2018 ACM International Joint Conference and 2018 International Symposium on Pervasive and Ubiquitous Computing and Wearable Computers, UbiComp ’18.

[3] Wang L., Gjoreski H., Ciliberto M., Lago P., Murao K., Okita T., Roggen D. (2019). Summary of the Sussex-Huawei Locomotion-Transportation Recognition Challenge 2019. Proceedings of the Adjunct Proceedings of the 2019 ACM International Joint Conference on Pervasive and Ubiquitous Computing and 2019 ACM International Symposium on Wearable Computers.

[4] Wang L., Gjoreski H., Ciliberto M., Lago P., Murao K., Okita T., Roggen D. (2020). Summary of the Sussex-Huawei Locomotion-Transportation Recognition Challenge 2020. Proceedings of the Adjunct Proceedings of the 2020 ACM International Joint Conference on Pervasive and Ubiquitous Computing and 2020 ACM International Symposium on Wearable Computers, UbiComp-ISWC ’20, Virtual.

[5] Gjoreski M., Janko V., Slapničar G., Mlakar M., Reščič N., Bizjak J., Drobnič V., Marinko M., Mlakar N., Luštrek M. (2020). Classical and deep learning methods for recognizing human activities and modes of transportation with smartphone sensors. Inf. Fusion.

[6] Sun B., Feng J., Saenko K. (2015). Return of Frustratingly Easy Domain Adaptation. arXiv.

[7] Kiprijanovska I., Gjoreski H., Gams M. (2020). Detection of gait abnormalities for fall risk assessment using wrist-worn inertial sensors and deep learning. Sensors.

[8] Luštrek M., Bohanec M., Cavero Barca C., Ciancarelli M.C., Clays E., Dawodu A.A., Derboven J., De Smedt D., Dovgan E., Lampe J. (2021). A Personal Health System for Self-Management of Congestive Heart Failure (HeartMan): Development, Technical Evaluation, and Proof-of-Concept Randomized Controlled Trial. JMIR Med. Inform..

[9] Kim H.G., Kim G.Y., Kim J.Y. (2019). Music Recommendation System Using Human Activity Recognition From Accelerometer Data. IEEE Trans. Consum. Electron..

[10] Patil P., Kumar K., Gaud N., Semwal V.B. Clinical Human Gait Classification: Extreme Learning Machine Approach. Proceedings of the 2019 1st International Conference on Advances in Science, Engineering and Robotics Technology (ICASERT).

[11] Jain R., Semwal V.B., Kaushik P. (2021). Deep ensemble learning approach for lower extremity activities recognition using wearable sensors. Expert Syst..

[12] Anagnostopoulou E., Urbančič J., Bothos E., Magoutas B., Bradesko L., Schrammel J., Mentzas G. (2020). From mobility patterns to behavioural change: Leveraging travel behaviour and personality profiles to nudge for sustainable transportation. J. Intell. Inf. Syst..

[13] Brazil W., Caulfield B. (2013). Does green make a difference: The potential role of smartphone technology in transport behaviour. Transp. Res. Part C Emerg. Technol..

[14] Wan S., Qi L., Xu X., Tong C., Gu Z. (2019). Deep learning models for real-time human activity recognition with smartphones. Mob. Networks Appl..

[15] Ronao C.A., Cho S.B. (2016). Human activity recognition with smartphone sensors using deep learning neural networks. Expert Syst. Appl..

[16] Ordóñez F.J., Roggen D. (2016). Deep convolutional and lstm recurrent neural networks for multimodal wearable activity recognition. Sensors.

[17] Zhao B., Li S., Gao Y. IndRNN based long-term temporal recognition in the spatial and frequency domain. Proceedings of the Adjunct 2020 ACM International Joint Conference on Pervasive and Ubiquitous Computing and 2020 ACM International Symposium on Wearable Computers.

[18] Zhu Y., Luo H., Chen R., Zhao F., Su L. DenseNetX and GRU for the Sussex-Huawei locomotion-transportation recognition challenge. Proceedings of the Adjunct 2020 ACM International Joint Conference on Pervasive and Ubiquitous Computing and 2020 ACM International Symposium on Wearable Computers.

[19] Wang L., Gjoreski H., Ciliberto M., Lago P., Murao K., Okita T., Roggen D. (2021). Three-Year Review of the 2018–2020 SHL Challenge on Transportation and Locomotion Mode Recognition From Mobile Sensors. Front. Comput. Sci..

[20] Cook D., Feuz K.D., Krishnan N.C. (2013). Transfer learning for activity recognition: A survey. Knowl. Inf. Syst..

[21] Bridle J., Cox S.J. (1990). Recnorm: Simultaneous normalisation and classification applied to speech recognition. Adv. Neural Inf. Process. Syst..

[22] Ghafoorian M., Mehrtash A., Kapur T., Karssemeijer N., Marchiori E., Pesteie M., Guttmann C.R., de Leeuw F.E., Tempany C.M., Van Ginneken B. (2017). Transfer learning for domain adaptation in mri: Application in brain lesion segmentation. Proceedings of the International Conference on Medical Image Computing and Computer-Assisted Intervention.

[23] Chattopadhyay R., Sun Q., Fan W., Davidson I., Panchanathan S., Ye J. (2012). Multisource domain adaptation and its application to early detection of fatigue. ACM Trans. Knowl. Discov. Data (TKDD).

[24] Csurka G. (2017). Domain adaptation for visual applications: A comprehensive survey. arXiv.

[25] Zhang Y., Nie S., Liu W., Xu X., Zhang D., Shen H.T. Sequence-to-sequence domain adaptation network for robust text image recognition. Proceedings of the IEEE/CVF Conference on Computer Vision and Pattern Recognition.

[26] Deng J., Zhang Z., Eyben F., Schuller B. (2014). Autoencoder-based unsupervised domain adaptation for speech emotion recognition. IEEE Signal Process. Lett..

[27] Sun S., Zhang B., Xie L., Zhang Y. (2017). An unsupervised deep domain adaptation approach for robust speech recognition. Neurocomputing.

[28] Gjoreski M., Kalabakov S., Luštrek M., Gams M., Gjoreski H. Cross-dataset deep transfer learning for activity recognition. Proceedings of the Adjunct Proceedings of the 2019 ACM International Joint Conference on Pervasive and Ubiquitous Computing and Proceedings of the 2019 ACM International Symposium on Wearable Computers.

[29] Too E.C., Yujian L., Njuki S., Yingchun L. (2019). A comparative study of fine-tuning deep learning models for plant disease identification. Comput. Electron. Agric..

[30] Borgwardt K.M., Gretton A., Rasch M.J., Kriegel H.P., Schölkopf B., Smola A.J. (2006). Integrating structured biological data by kernel maximum mean discrepancy. Bioinformatics.

[31] Sun F., Wu H., Luo Z., Gu W., Yan Y., Du Q. (2019). Informative feature selection for domain adaptation. IEEE Access.

[32] Jiang J., Zhai C. (2007). Instance weighting for domain adaptation in NLP. ACL.

[33] Reddy S., Mun M., Burke J., Estrin D., Hansen M., Srivastava M. (2010). Using mobile phones to determine transportation modes. ACM Trans. Sens. Networks.

[34] Khan A.M., Siddiqi M.H., Lee S.W. (2013). Exploratory data analysis of acceleration signals to select light-weight and accurate features for real-time activity recognition on smartphones. Sensors.

[35] Shi D., Wang R., Wu Y., Mo X., Wei J. (2017). A novel orientation-and location-independent activity recognition method. Pers. Ubiquitous Comput..

[36] Hachiya H., Sugiyama M., Ueda N. (2012). Importance-weighted least-squares probabilistic classifier for covariate shift adaptation with application to human activity recognition. Neurocomputing.

[37] Venkatesan A., Krishnan N.C., Panchanathan S. Cost-sensitive boosting for concept drift. Proceedings of the International Workshop on Handling Concept Drift in Adaptive Information Systems.

[38] Chapelle O., Schölkopf B., Zien A. (2009). Semi-Supervised Learning. IEEE Trans. Neural Netw..

[39] Guan D., Yuan W., Lee Y.K., Gavrilov A., Lee S. (2007). Activity recognition based on semi-supervised learning. Proceedings of the 13th IEEE International Conference on Embedded and Real-Time Computing Systems and Applications (RTCSA 2007).

[40] Mahdaviani M., Choudhury T. (2007). Fast and scalable training of semi-supervised crfs with application to activity recognition. Adv. Neural Inf. Process. Syst..

[41] Stikic M., Van Laerhoven K., Schiele B. (2008). Exploring semi-supervised and active learning for activity recognition. Proceedings of the 2008 12th IEEE International Symposium on Wearable Computers.

[42] Vo Q.V., Hoang M.T., Choi D. (2013). Personalization in mobile activity recognition system using K-medoids clustering algorithm. Int. J. Distrib. Sens. Networks.

[43] Kose M., Incel O.D., Ersoy C. Online human activity recognition on smart phones. Proceedings of the Workshop on Mobile Sensing: From Smartphones and Wearables to Big Data.

[44] Wang C., Xu Y., Liang H., Huang W., Zhang L. (2018). WOODY: A Post-Process Method for Smartphone-Based Activity Recognition. IEEE Access.

[45] Janko V., Gjoreski M., De Masi C.M., Reščič N., Luštrek M., Gams M. (2019). Cross-Location Transfer Learning for the Sussex-Huawei Locomotion Recognition Challenge. Proceedings of the Adjunct 2019 ACM International Joint Conference on Pervasive and Ubiquitous Computing and 2019 ACM International Symposium on Wearable Computers.

[46] Kalabakov S., Stankoski S., Reščič N., Kiprijanovska I., Andova A., Picard C., Janko V., Gjoreski M., Luštrek M. (2020). Tackling the SHL Challenge 2020 with Person-Specific Classifiers and Semi-Supervised Learning. Proceedings of the Adjunct 2020 ACM International Joint Conference on Pervasive and Ubiquitous Computing and 2020 ACM International Symposium on Wearable Computers, UbiComp-ISWC ’20, Virtual.

[47] Gjoreski H., Ciliberto M., Wang L., Morales F.J.O., Mekki S., Valentin S., Roggen D. (2018). The University of Sussex-Huawei locomotion and transportation dataset for multimodal analytics with mobile devices. IEEE Access.

[48] Banos O., Garcia R., Holgado-Terriza J.A., Damas M., Pomares H., Rojas I., Saez A., Villalonga C. (2014). mHealthDroid: A novel framework for agile development of mobile health applications. Proceedings of the International Workshop on Ambient Assisted Living.

[49] Banos O., Villalonga C., Garcia R., Saez A., Damas M., Holgado-Terriza J.A., Lee S., Pomares H., Rojas I. (2015). Design, implementation and validation of a novel open framework for agile development of mobile health applications. Biomed. Eng. Online.

[50] Anguita D., Ghio A., Oneto L., Parra Perez X., Reyes Ortiz J.L. A public domain dataset for human activity recognition using smartphones. Proceedings of the 21th International European Symposium on Artificial Neural Networks, Computational Intelligence and Machine Learning.

[51] Roggen D., Calatroni A., Rossi M., Holleczek T., Förster K., Tröster G., Lukowicz P., Bannach D., Pirkl G., Ferscha A. Collecting complex activity datasets in highly rich networked sensor environments. Proceedings of the 2010 Seventh International Conference on Networked Sensing Systems (INSS).

[52] Reiss A., Stricker D. (2012). Introducing a new benchmarked dataset for activity monitoring. Proceedings of the 2012 16th International Symposium on Wearable Computers.

[53] Zappi P., Stiefmeier T., Farella E., Roggen D., Benini L., Troster G. (2007). Activity recognition from on-body sensors by classifier fusion: Sensor scalability and robustness. Proceedings of the 2007 3rd International Conference on Intelligent Sensors, Sensor Networks and Information.

[54] Bannach D., Kunze K., Weppner J., Lukowicz P. Integrated tool chain for recording and handling large, multimodal context recognition data sets. Proceedings of the 12th ACM International Conference Adjunct Papers on Ubiquitous Computing-Adjunct.

[55] Department of Intelligent Systems, Jožef Stefan Institute Cr-Features. https://pypi.org/project/cr-features/.

[56] Janko V., Reščič N., Mlakar M., Drobnič V., Gams M., Slapničar G., Gjoreski M., Bizjak J., Marinko M., Luštrek M. (2018). A New Frontier for Activity Recognition: The Sussex-Huawei Locomotion Challenge. Proceedings of the 2018 ACM International Joint Conference and 2018 International Symposium on Pervasive and Ubiquitous Computing and Wearable Computers.

[57] Cvetković B., Szeklicki R., Janko V., Lutomski P., Luštrek M. (2018). Real-time activity monitoring with a wristband and a smartphone. Inf. Fusion.

